# Management of patients with acute ST-elevation myocardial infarction: Results of the FAST-MI Tunisia Registry

**DOI:** 10.1371/journal.pone.0207979

**Published:** 2019-02-22

**Authors:** Faouzi Addad, Abdallah Mahdhaoui, Jeridi Gouider, Essia Boughzela, Samir Kamoun, Mohamed Rachid Boujnah, Habib Haouala, Habib Gamra, Faouzi Maatouk, Ali Ben Khalfallah, Salem Kachboura, Hedi Baccar, Nejeh Ben Halima, Ali Guesmi, Khaled Sayahi, Wissem Sdiri, Ali Neji, Ahmed Bouakez, Sami Milouchi, Kais Battikh, Yves Jullieres, Nicolas Danchin, Jean Jacques Monsuez, Genevieve Mulak, Albert Hagege, Vincent Bataille, Rafik Chettaoui, Mohamed Sami Mourali, the FAST-MI Tunisia investigators

**Affiliations:** 1 Cardiology Departments: CHU Abderrahmen Mami, Ariana, Tunisia; 2 CHU Farhat Hached, Sousse, Tunisia; 3 CHU de Sahloul, Sousse, Tunisia; 4 CHU Hédi Chaker, Sfax, Tunisia; 5 CHU Mongi Slim, Marsa, Tunisia; 6 Hôpital Militaire Principal d’Instruction de Tunis, Tunis, Tunisia; 7 Cardio A CHU Fattouma Bourguiba, Monastir, Tunisia; 8 Cardio B CHU Fattouma Bourguiba, Monastir, Tunisia; 9 Hôpital de Menzel Bourguiba, Menzel Bourguiba, Tunisia; 10 Hôpital Charles Nicolles, Tunis, Tunisia; 11 Hôpital régional Ibn El Jazzar, Kairouan, Tunisia; 12 Hôpital régional Mohamed Ben Sassi, Gabes, Tunisia; 13 Hôpital régional M’Hamed Bourguiba, Kef, Tunisia; 14 Hôpital régional Habib Bougatfa, Bizerte, Tunisia; 15 Hôpital régional Ben Guerdene, Medenine, Tunisia; 16 Hôpital régional Jendouba, Jendouba, Tunisia; 17 Hôpital régional de Medenine, Medenine, Tunisia; 18 Clinique de Djerba, Djerba, Tunisia; 19 Département de Cardiologie, CHU Nancy, Nancy, France; 20 APHP, Hôpital Européen Georges Pompidou, Paris, France; 21 APHP Hôpital R Muret, Hôpitaux Universitaires de Paris Seine Saint Denis, Sevran, France; 22 Société Française de Cardiologie, Paris, France; 23 Service d’aide médicale urgente, CHU Toulouse 3, Toulouse, France; 24 Clinique de Tunis, Tunis, Tunisia; 25 CHU La Rabta, Tunis, Tunisia; Azienda Ospedaliero Universitaria Careggi, ITALY

## Abstract

**Background:**

The FAST-MI Tunisia registry was set up by the Tunisian Society of Cardiology and Cardiovascular Surgery to assess the demographic and clinical characteristics, management and hospital outcome of patients with ST-elevation myocardial infarction (STEMI).

**Methods:**

Data for 459 consecutive patients (mean age 60.8 years; 88.5% male) with STEMI, treated in 16 public hospitals (representing 72.2% of public hospitals in Tunisia treating STEMI patients), were collected prospectively.The most common risk factors were smoking (63.6%), hypertension (39.7%), diabetes (32%) and dyslipidaemia (18.2%).

**Results:**

Among the 459 patients, 61.8% received reperfusion therapy: 30% with primary percutaneous coronary intervention (PPCI) and 31.8% with intravenous fibrinolysis (IF) (28.6% with pre-hospital thrombolysis). The median time from symptom onset to thrombolysis was 185 min and to PPCI was 358 min. In-hospital mortality was 5.3%. Compared with those managed at regional hospitals, patients managed at interventional university hospitals (*n* = 357) were more likely to receive reperfusion therapy (52.9% vs. 34.1%; p<0.001), with less IF (28.6% vs. 43.1%; p = 0.002) but more PPCI (37.8% vs. 3.9%; p<0.0001). However, in-hospital mortality in the two types of hospitals was similar (5.3% vs. 5.1%; p = 0.866).

**Conclusions:**

Data from the FAST-MI Tunisia registry show that a pharmaco-invasive strategy of management for STEMI should be promoted in non-interventional regional hospitals.

## Introduction

Cardiovascular diseases represent the main cause of mortality in Tunisia [1,2]. During the last decade, the management of acute coronary syndromes (ACSs) has dramatically improved, particularly in the setting of ST elevation myocardial infarction (STEMI), leading to important reduction in hospital mortality, and to better mid- and long-term cardiovascular outcome [3].

The treatment strategies of ACSs are very heterogeneous from one country or one region to another, and are directly linked to the presence or not of an urgent medical aid service (UMAS) and the proximity of a catheterization laboratory determining the feasibility of primary percutaneous coronary intervention (PPCI) [4].

The FAST-MI French registry, which included patients admitted for acute myocardial infarction (AMI) at different time periods, revealed an improvement in the management of such a critical condition with a significant decrease in mortality between 1995 and 2010 [5]. This decrease in mortality was strongly linked to a greater use, and more rapid access to primary PCI, but also to change in patient profile, early use of recommended medications, and general organisation of care [5].

Since 1980, UMAS has covered 50% of Tunisia (including 75% of Tunisian population) with human and equipment ressources providing the possibility to perform pre-hospital intravenous fibrinolysis (IF), and 30 cardiac cath labs (mainly in the coastal regions) are currently available allowing a rapid management of patients admitted for STEMI.

However, data regarding the management of patients referred for ACS in Tunisia are scarcely reported. The Access-Tunisia registry, initiated between 2007 and 2008, included a limited number of patients only enrolled at university hospitals [6].

Therefore, we sought to analyse the demographic and the clinical characteristics as well as the modalities of myocardial reperfusion employed in STEMI patients enrolled into the FAST-MI Tunisia registry, comparing the management strategies between university (generally with cath lab) and regional hospitals (without cath lab), and investigating the independent predictors of in-hospital mortality.

## Methods

### FAST-MI registry and participating centers

**Fig 1:**
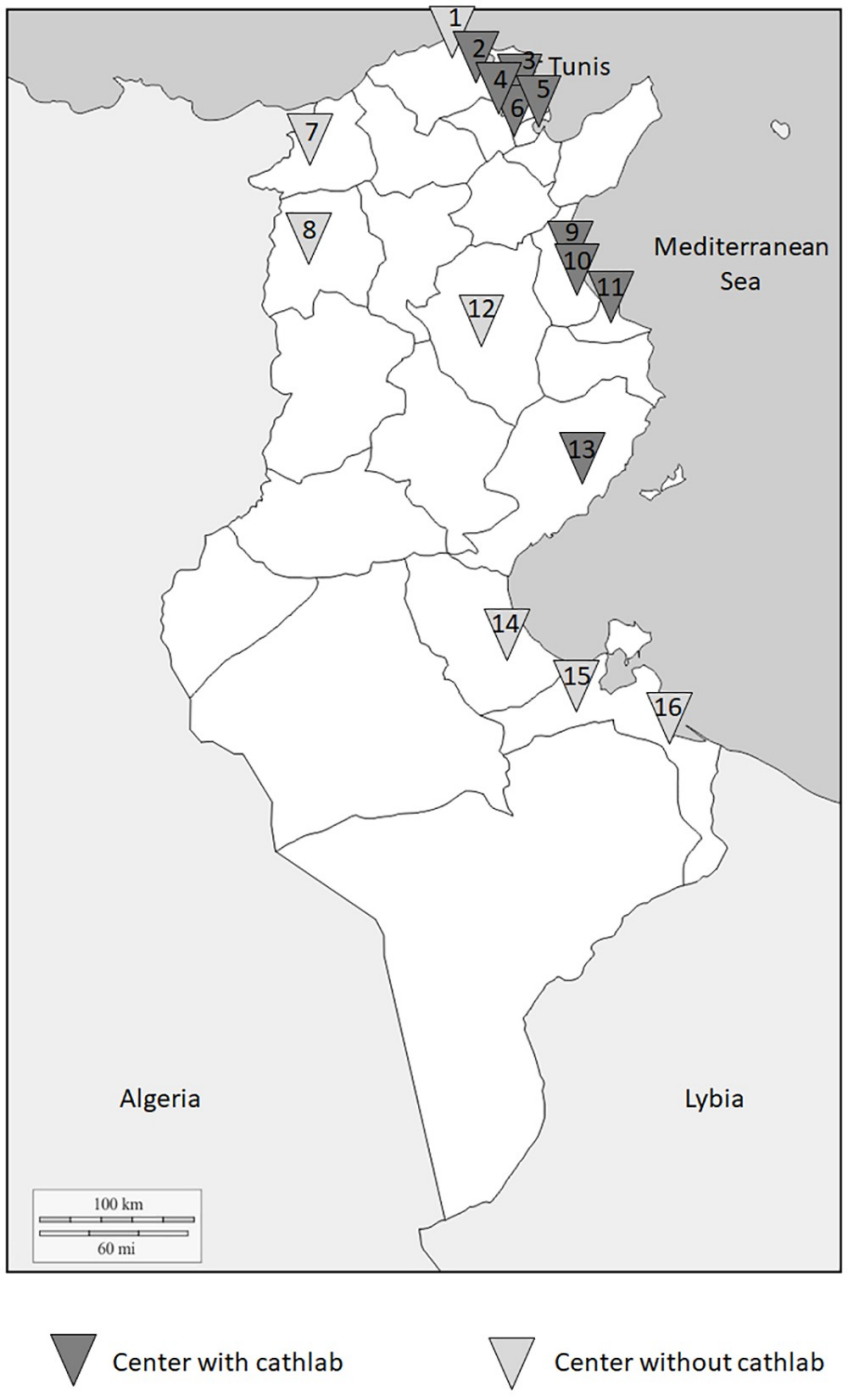
Geographical map of participating centers. 1) Habib Bougatfa Hospital-Bizerte; 2) Menzel Bourguiba Hospital-Bizerte; 3) Abderrahmen Mami Hospital-Ariana; 4) Mongi Slim Hospital- Marsa-Tunis; 5) Military Hospital-Tunis; 6) Rabta Hospital-Tunis; 7) Jendouba Hospital-Jendouba; 8) M’hamed Bourguiba Hospital-Kef; 9) Sahloul Hospital-Sousse; 10) Farhat Hached Hospital-Sousse; 11) Fattouma Bourguiba Hospital-Monastir; 12) Ibn El Jazzar Hospital-Kairouan; 13) Hedi Chaker Hospital-Sfax; 14) Mohamed Ben Sassi Hospital- Gabes; 15) Habib Bourguiba Hospital-Medenine; 16) Ben Guerdene Hospital- Medenine.

The FAST-MI Tunisia registry was set up through a close collaboration between the Tunisian Society of Cardiology and Cardiovascular Surgery (TSCCS) and the French Society of Cardiology (FSC). Participation in this first Tunisian registry was proposed to all Tunisian hospitals (in both public and private sectors). Sixteen public hospitals (either university or regional hospitals) and one private centre (including only 2 patients) participated in the study. Only patients enrolled at public hospitals were retained for the analysis. This registry included almost 3/4 (72.2%) of Tunisian public hospitals (16 out of 22 centers) managing STEMI patients in different regions of Tunisia: 10 university hospitals and 6 regional hospitals. Fig 1. illustrates the geographical distribution of the participating centers. The preliminary results of patients enrolled during the first three months of the study period were previously reported [7].

### Study population

Between September 2014 and March 2015, consecutive patients with STEMI were prospectively enrolled into the FAST-MI Tunisia registry.

Each patient had to meet the following criteria: i) man or woman aged 18 years or over, ii) ST elevation ≥1 mm was seen in at least two contiguous leads in any location on the index or qualifying ECG, or when presumed new left bundle branch block or documented new Q waves were observed. Patients admitted after resuscitation of a cardiac arrest were included only if the cardiac arrest had been preceded by chest pain suggestive of AMI. Patients in whom the diagnosis of AMI was refuted in favour of alternative diagnoses (such as acute myocarditis) were excluded.

### Ethics statement

The study protocol was approved by the Ethics committee of the Tunisian Society of Cardiology and Cardiovascular Surgery. An informed consent was obtained in all patients; and the study was carried out according to Helsinki declaration.

### Data collection

In each participating center, demographic and clinical data, as well as the management strategies in all patients referred to cardiology intensive care units (CICU) for STEMI within 48h from symptoms onset were collected. ECG interpretation was performed by the enrolling physician in each participating center. Data collection was performed via an electronic case record form, identical to that used in the FAST-MI French study [7].

### Statistical analysis

Continuous variables were presented as mean ±standard deviations and were compared using one-way analysis of variance, or as median and interquartile range, as appropriate. Categorical variables were presented as counts and percentages and compared with the Chi ^2^ test when appropriate (expected frequency>5). Otherwise, the Fisher exact test was used. The predictors of in-hospital mortality were identified using a binary logistic regression analysis. All variables were tested in univariate analyses. Univariate variables with a p value <0.1 were included in a statistical model in order to detect the independent predictors using multivariate regression analysis with the Wald method. In all cases, p values <0.05 were considered statistically significant. All data were processed using the Statistical Package for Social Sciences, version 20 (SPSS, Chicago, IL, USA)

## Results

### Characteristics of the study population

**Fig 2:**
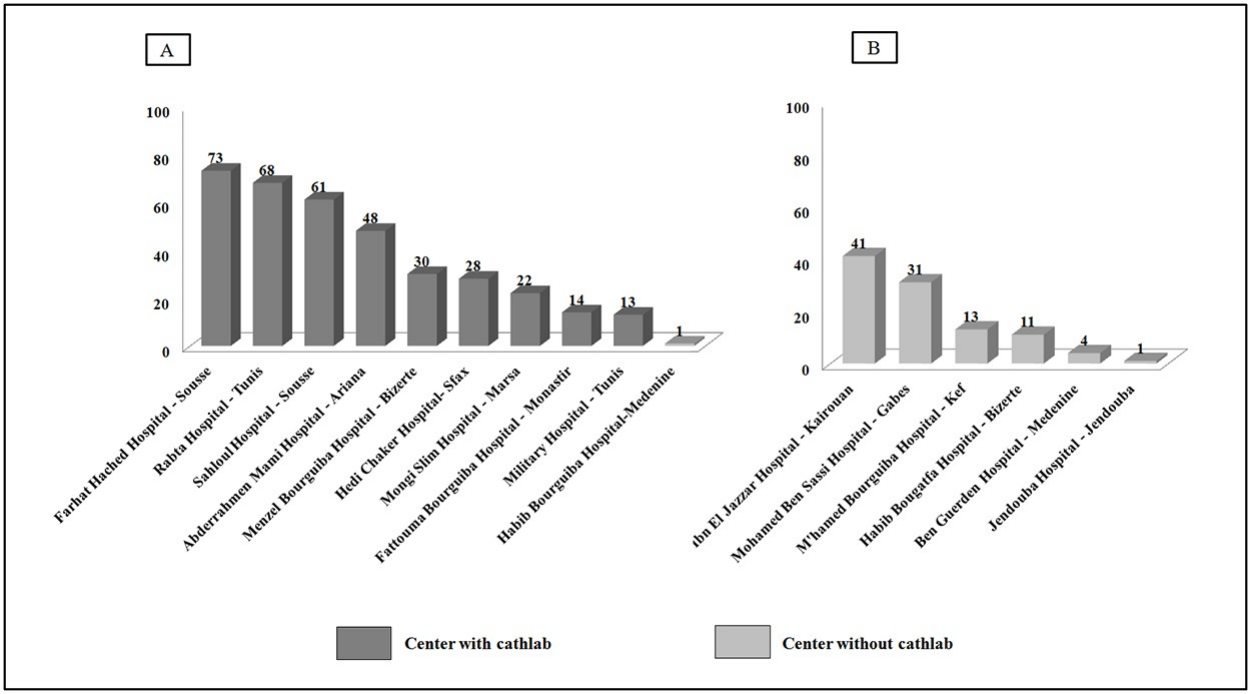
Patients’ enrolment in different participating centers. A) University hospitals; B) Regional hospitals.

A total of 459 consecutive STEMI patients were prospectively enrolled into FAST-MI Tunisia registry. Patients’ distribution according to the different participating Tunisian centers is shown in Fig 2. Nine centers (all of them university hospitals) were equipped with a cath lab providing primary PCI. The majority of patients (78%) were enrolled at university hospitals.

The mean age of the study population was 60.8 ± 12.2 years (range, 27–87 years) and the majority (88.5%) were male. Fifteen percent were older than 75 years, while 6.6% were younger than 45. The prevalence of risk factors was as follows: smoking (63.6%), hypertension (39.7%), diabetes mellitus (32%) and dyslipidaemia (18.2%).

Except higher prevalence of female gender (27.5% vs.16%; p = 0.04) and lower prevalence of smoking (48.9% vs. 67.5%; p = 0.01) in regional hospitals in comparison with university hospitals, no other significant differences were observed in terms of clinical baseline characteristics (Table 1).

**Table 1:** Baseline characteristics of the study population.

	All patients (N = 459)	University Hospitals (N = 357)	Regional Hospital (N = 102)	P value
Age, years, mean ± SD	60.8 ± 12.2	60.5 ± 12.3	61.7 ± 11.9	ns
Male gender, n (%)	374 (81.5)	300 (84)	74 (72.5)	**0.04**
Active smoking, n(%)	279 (63.6)	233 (67.5)	46 (48.9)	**0.01**
Diabetes, n(%)	147 (32)	121 (33.9)	26 (25.5)	ns
Hypertension, n(%)	174 (39.7)	131 (37.8)	43 (47.3)	ns
Hypercholesterolemia, n(%)	75 (18.2)	61 (18.8)	14 (15.9)	ns
**Clinical presentation**				ns
Chest pain, n(%)	400 (91.5)	309 (90.6)	91 (94.8)	
Acute heart failure ≥ Killip 2	46 (11.4)	34 (10.3)	12 (16.7)	
Syncope, n(%)	15 (3.7)	6 (1.8)	9 (12.3)	
Resuscitated cardiac arrest, n(%)	6 (1.5)	5 (1.5)	1 (1.4)	
Heart rate, bpm, mean ± SD	84.9 ± 26.5	85.2 ± 27.5	83.4 ± 18.3	ns
SBP, mmHg, mean ± SD	130.8 ± 27.4	129.3 ± 26.2	140.5 ± 33.8	ns
**Electrographic characteristics**				
Sinus rhythm, n (%)	422 (97.7)	335 (97.4)	87 (98.9)	ns
3^rd^ degree AVB, n(%)	15 (4)	12 (3.9)	3 (4.4)	ns
ST-elevation, n(%)	416 (94.3)	333 (94.3)	83 (94.3)	ns
*Anterior leads*, *n(%)*	*232 (52*.*1)*	*177 (51*.*2)*	*55 (55*.*6)*	
*Inferior leads*, *n(%)*	*186 (41*.*8)*	*146 (42*.*2)*	*40 (40*.*4)*	
LBBB, n(%)	8 (1.8)	8 (2.3)	0	ns
Q wave, n(%)	199 (48.3)	158 (47.7)	41 (50.6)	ns

Abbreviations: AVB = auriculo-ventricular block; bpm = beats per minute; LBBB = left bundle branch block; ns = non significant (p<0.05); SBP = systolic blood pressure.

The main symptoms observed at first medical contact were the following: chest pain (91.5%), acute heart failure (11.4%), syncope (3.7%) and resuscitated cardiac arrest (1.5%). Baseline clinical and electrocardiographic characteristic are summarised in Table 1.

Regarding transfer conditions, only 85 patients (18.5%) were managed by UMAS at initial medical contact, and all of them were transported to university hospitals. Patients were admitted at emergency department in 54% of cases, at CICU in 32% and directly at cath lab in 12.8%. Only 46% of patients presented within 3 h of symptom onset.

### Reperfusion modalities and management strategies

IF was administered in 31.8% of patients [tenecteplase (62.2%), streptokinase (32.8%) and alteplase-rt-Pa (5%)]. Pre-hospital IF in UMAS ambulance was performed in 27.7% of IF cases. The median delay between symptom onset and IF was 180 min [IQR: 120–285 min]. Rescue PCI angioplasty was performed in 6.6% of cases.

Primary PCI was performed in 30% of patients; after a median delay of 360 min [IQR: 204–570 min] from symptom onset. Radial and femoral approaches were used in 61% and 39%, respectively. GPIIbIIIa inhibitors (tirofiban in all cases) were administered in 33.8% of patients undergoing primary PCI; while thrombo-aspiration was performed in 46.7% of cases. In patients managed with primary PCI, drug-eluting stents were implanted in 35.7% of cases, while bare-metal stents were used in the remaining 64.3% of patients. Primary PCI was judged successful in 91.2% of cases; whereas, a final TIMI flow grade 0 or 1 was reported in 5.6% of cases.

Patients admitted for STEMI in regional hospitals received more frequently IF as compared to those admitted at university hospitals (43.1% vs. 28.6%; p = 0.002). Primary PCI was performed in only four patients (3.9%) referred to regional hospitals [vs. 37.8% in the university hospitals (p<0.001)].

In the whole cohort, coronary angiography was performed in 84.7% of patients (either initially or secondarily); more often in patients admitted at university hospitals than those at regional hospitals (93.2% vs. 54.9%; p<0.001). Normal coronary angiography was found in only 2% of cases. PCI (either primary or rescue or secondary) was performed in 66.2%, and coronary artery bypass grafting was indicated in 2.2% of cases.

Almost one-third of patients (31.6%) did not have any revascularization of the culprit artery; the majority of them in regional hospitals (52.9% vs. 34.1%; p<0.001). This was mainly due to an important delay at initial presentation [>24 hours (49.5% of all cases, and 66% in regional hospitals)].

During hospital stay, aspirin was prescribed in all cases and clopidogrel in 92.4% of cases. Unfractionated heparin was administered in 66.5% of cases, while the remaining 33.5% received low molecular weight heparin (enoxaparin in all cases).

### In-hospital outcome

Table 2 summarizes the in-hospital outcome of the study population. In-hospital death occurred in 24 patients (5.3%), and was similar between university and regional hospitals (5.3% vs. 5.1%; p = 0.866). On multivariate analysis, the presence of acute heart failure ≥ Killip class 2 at admission was the only independent predictor of in-hospital mortality [Odds ratio (OR) 18.1; 95%CI: 5.4–61.1; p<0.001].

**Table 2:** In-hospital outcome of the study population.

	All patients (N = 459)	University Hospitals (N = 357)	Regional Hospitals (N = 102)	P value
Death, n(%)	24 (5.5)	19 (5.6)	5 (5.4)	ns
Atrial fibrillation, n (%)	25 (6)	23 (7)	2 (2.3)	ns
Ventricular tachycardia, n(%)	11 (2.6)	9 (2.7)	2 (2.3)	ns
Ventricular fibrillation, n(%)	13 (3.1)	11 (3.3)	2 (2.3)	ns
Ischemia recurrence, n(%)	18 (4.3)	11 (3.3)	7 (8)	ns
Pericardial effusion, n(%)	9 (2.2)	7 (2.2)	2 (2.3)	ns
Stroke/TIA, n(%)	4 (0.9)	4 (1.1)	0	ns
**Mechanical complications**				ns
Significant ischemic MR, n(%)	2 (0.5)	2 (0.6)	0	
Free wall rupture, n(%)	1 (0.2)	1 (0.3)	0	
VSD, n(%)	2 (0.5)	1 (0.3)	1 (1.3)	
Major bleeding, n(%)	5 (1.2)	3 (0.9)	2 (2.3)	ns

Abbreviations: MR = mitral regurgitation; TIA = transient ischemic attack; VSD = ventricular septal defect.

## Discussion

The current study represents the first Tunisian registry including MI patients managed in public sector, in both university and regional hospitals. The FAST-MI Tunisia registry, initiated by the TSCCS, aimed to answer several questions about the epidemiology, management strategies and the immediate outcome of patients treated for STEMI in 2015.

In Tunisia, cardiovascular diseases are the leading causes of death (30%), 70% of those are due to coronary artery disease (CAD) [1]. Saidi et al. [1] reported that CAD mortality rates increased by 11.8% for men and 23.8% for women, between 1997 and 2009. Importantly, the optimization of initial MI treatment prevented about 90 deaths in 2009 [1].

As compared with the FAST-MI French registry [8], the mean age of Tunisian patients was younger (60 vs. 63 years); this was due not only to the demographic characteristics of the Tunisian population (mean age 31 years in the 2014 census), but also to the higher prevalence of cardiovascular risk factors such as active smoking (64% vs. 40%) and diabetes (32% vs. 17%). Indeed, it is known that these two risk factors are particularly associated with AMI in younger patients [9]. Conversely, the prevalence of hypertension (40% vs. 48%) and hypercholesterolemia (18% vs. 40%) was lower because these two factors are more frequently observed in an older population. Indeed, only 15% of our patients were aged >75 years compared to 48% in the French registry [8].

A decrease in the age of AMI patients was observed in the FAST-MI French study between 1995 and 2010 (66 vs. 63 years), this was mainly linked to a greater proportion of patients aged <60 years, in particular females in whom the prevalence of active smoking was considerably higher (37% to 73% in the case of AMI). The increasing prevalence of risk factors in our population (notably female smoking) as well as ageing of the Tunisian population suggest an increase in AMI prevalence in the future[1, 2]. Thus, it is important to reconsider prevention strategies in the general population particularly targeting alimentary habits and lifestyle.

Recent data in the literature have confirmed a decrease in mortality of patients admitted for STEMI over the past 15 years [5, 10–16]. This has mainly been attributed to the development of reperfusion therapies, the more frequent use of primary angioplasty and recommended medications from an early stage, and also to reduced treatment delays. Data from the FAST-MI Tunisia registry demonstrated the great heterogeneity in management of patients depending on the location and the type of hospital where they were managed. We observed a much greater use of thrombolysis in regional hospitals, because of the difficulty to transfer patients within 90 min to a PCI-capable center. Conversely in university hospitals, primary PCI was more frequently performed. Furthermore, there were significantly more patients admitted initially at regional hospitals, who did not benefit from any type of reperfusion.

If we refer to the data from the Access-Tunisia registry [6], which was conducted between 2007 and 2008 over a period of 13 months (*n* = 173 patients) including patients only in university hospitals, the use of primary angioplasty has considerably increased (21.9% vs. 37.8%). This demonstrates greater adherence to recommendations and better organization of the different stakeholders involved in the management of AMI patients particularly in big cities covered by UMAS with at least one PCI-capable center. Nevertheless, we observed that the role of UMAS was still insufficient even at university hospitals with a low level of pre-hospital IF (less than one-third of cases) and low number of patients directly referred to cath lab. In comparison with data from the FAST-MI French registry [5], the use of primary angioplasty in Tunisian university hospitals remains low. In addition, many insufficiencies in the management of patients admitted for STEMI were observed in regional hospitals, essentially related to a poorly structured healthcare network. Thrombolysis, the only strategy available on site, was used in less than one-half of cases and this was mostly related to late delay from first medical contact. One-quarter of patients received IF within <120 min. Tenecteplase was the most frequently used fibrinolytic agent and this was in accordance with the latest studies and recommendations in order to reduce the morbi-mortality and infarct size [17]. However, we also observed a very low use of pharmaco-invasive strategies despite very encouraging results [18,19], due to the fact that only 4.7% of patients were transferred from regional to university hospitals providing PCI facilities.

In terms of early mortality, the results of the FAST-MI French registry demonstrated a spectacular decrease in 30-day mortality, from 13.7% in 1995 to 4.4% in 2010 (reduction of 68%) [5]. This decrease in mortality was explained by the change in the population suffering an AMI (much younger by 3 years on average), shorter delays in management (120 min to 74 min), a greater involvement of UMAS (from 23% to 49%) and a greater use of primary angioplasty (49% in 1995 to 75% in 2010). The in-hospital mortality in our registry was 5.3%, which is slightly higher than in the French 2010 registry. Despite higher rate of primary PCI at university hospitals, the in-hospital outcome was similar to that observed at regional hospitals. This fact was probably due to longer delays for primary PCI in comparison with IF.

Hence, the question of how in-hospital mortality due to AMI can be further reduced in Tunisia? First, it is imperative to reduce the delays in management STEMI patients, which were 2-times longer in our registry, by increasing the awareness of the general public about the need to call UMAS/MERS (mobile emergency resuscitation service) promptly in case of chest pain. If IF is carried out within 2 hours, it provides a similar or even greater benefit than primary angioplasty [20–22]. Thus, regional hospitals should be encouraged to rapidly transfer patients, particularly the most serious ones, as soon as IF is started in the context of a pharmaco-invasive strategy, which might help to improve the prognosis [23,24]. A more important role for UMAS/MERS is fundamental in this context to optimize the management network.

### Study limitations

This study has several limitations. First, the sample size was relatively limited particularly in the group of patients referred to regional hospitals. Second, only data from public hospitals were analysed, this does not necessarily reflect the management of STEMI patients in private institutions (including 2/3 of Tunisian cath labs). Third, only clopidogrel was used, being the only P2Y12-receptor antagonist available in Tunisia. Fourth, information addressing ECG time and first medical contact to balloon time in PPCI cases, are missing. Finally, mid-and long-term outcome were beyond the scope of this study.

Nonetheless, the study represents the largest source of data patients hospitalised for STEMI in Tunisia so far, and it included patients consecutively over a 6-month period of time.

## Conclusions

Compared to previous data obtained in more selected centers, we observed a considerable improvement in the quality of management of STEMI patients in Tunisia. However, many insufficiencies were also highlighted by this registry, particularly among patients admitted at regional hospitals. The therapeutic strategy of management may be optimized by implementing an immediate « infarct plan » promoting medical transport, the use of hospitals dedicated to MI management, pre-hospital fibrinolysis and, finally, primary angioplasty in the context of a pharmaco-invasive strategy.

## Abbreviations

ACS: acute coronary syndrome
CAD: coronary artery disease
CICU: cardiology intensive care unit
FSC: French Society of Cardiology
IF: intravenous fibrinolysis
MERS: mobile emergency resuscitation service
OR: Odds ratio
PCI: percutaneous coronary intervention
STEMI: ST-elevation myocardial infarction
TSCCS: Tunisian Society of Cardiology and Cardiovascular Surgery
UMAS: urgent medical aid service
